# Cochlear implantation and cognitive function in the older adult population: current state of the art and future perspectives

**DOI:** 10.1016/j.bjorl.2024.101544

**Published:** 2025-01-28

**Authors:** Tinne Vandenbroeke, Ellen Andries, Marc J.W. Lammers, Anouk Hofkens-Van den Brandt, Griet Mertens, Vincent Van Rompaey

**Affiliations:** a Antwerp University Hospital (UZA), Department of Otorhinolaryngology, Head and Neck Surgery, Edegem, Belgium; b University of Antwerp, Faculty of Medicine and Health Sciences, Department of Translational Neurosciences, Resonant Labs Antwerp, Antwerp, Belgium

**Keywords:** Cochlear implant, Hearing, Cognition, Ageing

## Abstract

**Objectives:**

Hearing loss is associated with increased cognitive decline and incident dementia in older adults. Given the rapidly rising incidence of dementia, management of modifiable risk factors such as hearing loss, is essential to mitigate the impact on the individual and society in general. In this narrative review, we discuss the current state-of-art with respect to studying cognitive function before and after cochlear implantation in the elderly population. Future perspectives are considered to shed light into the pathophysiology of cognitive decline due to hearing loss and how objective measures may contribute to this field.

**Methods:**

The existing literature on cochlear implantation and cognition is reviewed. The potential limitations and objective measures for cognitive functioning are discussed. Relevant studies were identified through a broad search of academic databases, including Pubmed and Web of Science. Given the narrative nature of this review, no strict inclusion or exclusion criteria were applied, allowing for a broad overview of the current state-of-the-art literature on the effects of cochlear implantation on cognitive functioning, the challenges of studying cognition in this population and future research directions.

**Results:**

Multiple studies have been able to demonstrate an improvement of cognitive functioning in older adults with severe-to-profound hearing loss after cochlear implantation. However, it is important to consider the challenges of studying cognition in this population. Evoked response potentials might have potential as an objective marker for cognition in this study population.

**Conclusion:**

Although there is no standardized study protocol to investigate cognition after cochlear implantation, a significant improvement in cognition is observed in the majority of studies one year after cochlear implantation. The identification of an objective marker of cognitive functioning will help unravel how cochlear implantation affects cognition.

## Highlights

• Use of cochlear implants can attenuate cognitive decline in adults with hearing loss.
• The cognitive test should be adapted for individuals with hearing loss.
• Evoked response potentials might serve as an objective marker for cognition.

## Introduction

Currently, around 20% of the world population is affected by hearing loss, and it is expected that these numbers will continue to increase. According to the World Health Organization (WHO), it is expected that by 2050 1 in 4 individuals will have hearing problems.^1^ It is well known that hearing loss has a considerable impact on individuals’ daily life, untreated hearing loss can lead to communication problems, social isolation, loneliness, and higher anxiety and depression rates.^2^, ^3^, ^4^ Moreover, an accelerated cognitive decline is observed in older adults with peripheral hearing impairment compared to normal hearing individuals, Lin et al., (2013) indicated a 24% increased risk of cognitive impairment in this population.^5^ This suggests that peripheral hearing impairment is associated with a higher dementia risk in later life and it might be a potential modifiable risk factor for the development of dementia.^5^, ^6^, ^7^

**Fig. 1:**
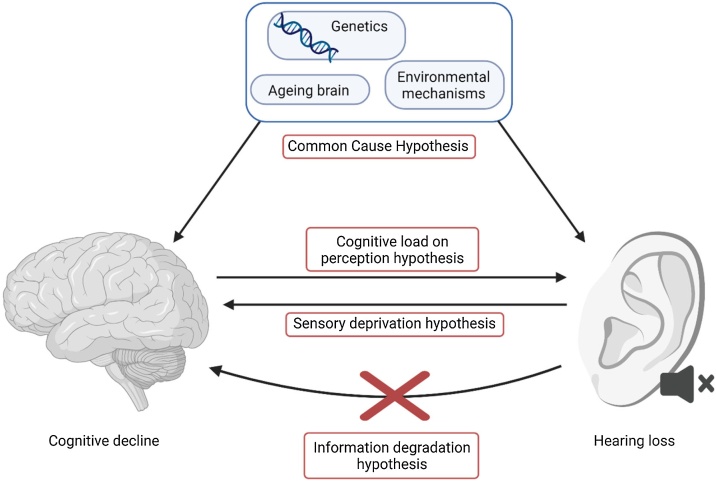
Proposed hypotheses for the underlying relation between hearing impairment and cognitive decline. Created with BioRender.com.

The following hypotheses have been proposed and studied concerning the underlying relationship between cognition and peripheral hearing loss: (1) Common cause hypothesis, (2) Cognitive load on perception hypothesis, (3) Sensory deprivation hypothesis, and (4) Information degradation hypothesis.^8^, ^9^, ^10^ According to the first hypothesis, hearing loss and cognitive decline both result from a similar factor, such as the ageing brain, environmental mechanisms or genetic factors. The cognitive load on perception hypothesis suggests that cognitive decline affects sensory processing, seemingly ‘simple’ sensory tasks increase in cognitive complexity and demand due to the cognitive decline. The sensory deprivation hypothesis suggests that sensory deprivation causes cognitive decline, as long-term hearing loss may reduce intellectually stimulating interactions with the environment, which can eventually lead to a cognitive decline. The final hypothesis, the information degradation hypothesis, suggests a similar relationship, but suggests that the impact on cognition is reversible. This implies that compensating for perceptual difficulties, for instance with a Cochlear Implant (CI), may also improve cognitive performance (Fig. 1). Currently, the underlying relationship between peripheral hearing loss and cognition is unknown.

Hearing loss can be treated with various hearing technologies, such as hearing aids and CIs. Lin et al., (2023) performed a multicenter, randomized controlled trial in older adults to investigate the effect of hearing intervention on cognitive functioning. They indicated that a hearing aid might reduce cognitive decline in older adults with an increased risk of cognitive decline, but not in the population with a reduced risk of cognitive decline.^11^

In this review, we aim to provide a clear overview of the current state-of-the-art literature with respect to studying cognitive function before and after cochlear implantation in the elderly population. Moreover, we will go over the limitations of studying cognitive functioning in a CI population and discuss how objective measures for cognitive functioning may contribute to this field.

## How cochlear implantation has an impact on older adults with severe-to-profound sensorineural hearing loss

CIs are surgically implantable devices that directly stimulate the auditory nerve and avoid damaged parts of the ear, e.g., damaged or missing hair cells. They are used in individuals with severe-to-profound hearing loss when a conventional hearing aid cannot be used or only has limited benefit.^12^ Previous research indicated a positive effect of hearing restoration on Health-Related Quality of Life (HRQoL) using different questionnaires.^13^, ^14^, ^15^, ^16^, ^17^, ^18^

### Effect on cognitive function

In addition to the positive effect on HRQoL, many studies demonstrated that the use of a CI attenuates the cognitive decline observed in individuals with hearing loss.^13^, ^14^, ^16^, ^17^ Currently, there is a wide range of cognitive assessment tools available and each tool has its advantages and disadvantages. One very important aspect is the suitability of the cognitive test to access cognition in individuals with hearing loss. This is important to avoid bias in the results caused by their hearing loss, as items that are presented only aurally may not be well perceived or may be misperceived by the individual. Hence, modifications of the original assessment tools are essential to study the effect of CI on cognition and not the increase in performance due to improved hearing.^13^, ^19^ Most studies provided written instructions to the subject during the cognitive assessment to avoid improvement in cognitive performance linked to improvement in hearing. The variability in cognitive assessment tools and the absence of a standardized study protocol to study the effect of CI on cognition makes comparison between different study results difficult. However, in the majority of studies, an improvement in overall cognitive functioning was observed after cochlear implantation using different cognitive evaluation tools, including Mini-Mental State Examination (MMSE),^14^, ^20^ Repeatable Battery for the Assessment of Neuropsychological Status for hearing impaired individuals (RBANS-H),^13^, ^17^ multi-modular computer-based test battery (ALAcog).^16^, ^21^ A longitudinal controlled study was performed by Mertens et al., (2021) to investigate the effect of CI on cognition. An intervention group was matched to a control group based on the following criteria: age, education, and percentage of residual hearing in the best ear. This control group allowed correction for practice effects linked to the repeated testing. One year after cochlear implantation a significant improvement in the total RBANS-H score and the subdomain “Attention” was observed. However, these results were still worse compared to normal-hearing individuals.^17^ Sonnet et al., (2017) were not able to identify a meaningful effect of cochlear implantation on global cognitive functioning using the MMSE,^18^ this can be explained by the fact that screening tests are less sensitive and only differentiate between normal and abnormal cognitive functioning. However, other studies did find a significant improvement in cognitive functioning one year after cochlear implantation using the MMSE.^14^, ^20^ A recent study demonstrated that the positive effect of cochlear implantation on cognition is not only present in individuals with normal cognitive scores preoperatively but also in individuals with Mild Cognitive Impairment (MCI).^22^

When comparing the effect of cochlear implantation on the cognitive subdomains some minor differences can be observed between the studies. Some studies used the RBANS-H, which is corrected for age and investigates five different cognitive domains, while other studies made their own selection of cognitive tests. A study by Völter et al., (2018) found significant improvements in executive functions such as attention, inhibition, and working memory. Additionally, for delayed recall, and long-term memory a significant change was observed.^16^ The results of Claes et al., (2018) concur with these findings, using the RBANS-H they found significant effects in the cognitive domains Attention, Immediate Memory, and Delayed Memory one year after cochlear implantation.^13^ However, in comparison to the study of Mertens et al., (2021), these studies did not include a control group, so they were not able to correct for possible practice effects.^13^, ^16^, ^17^ A study by Mosnier et al., (2015) describes the effect of cochlear implantation on cognition in a group of individuals with a poor cognitive score preoperatively, and in individuals with the best cognitive performance. They observed an improvement in cognitive functioning after cochlear implantation in over 80% of the subjects who had a low cognitive score before implantation. Subjects with a high cognitive score preoperatively remained stable after cochlear implantation, and around 24% showed a slight decline.^14^ It seems that the greatest effect on cognition is present in individuals with a lower score preoperatively, and that the effect is less pronounced in the high performers. Nevertheless, solid evidence is lacking as most studies investigate only the poor performers or the group of poor and high performers as a whole. To make valuable conclusions regarding the difference in cognitive evolution, the effect of cochlear implantation on cognition needs to be investigated in a group of poor performers, and high performers.

### Does the effect on cognitive function last?

Few research groups investigated the long-term effect of CI on cognition. Studies that have been performed on this topic confirm previous findings, i.e., significant cognitive improvement one year after cochlear implantation, but they also showed that this effect stabilizes over time.^20^, ^21^ Völter et al., (2022) investigated the long-term effect of cochlear implantation on cognition using a computer-based neurocognitive assessment tool consisting of nine subtests covering different cognitive domains. Cognitive evaluation of the subjects took place at three timepoints; preoperatively, 12-months, and up to 65-months after cochlear implantation. A significant improvement was found between the first two timepoints on the following cognitive domains; attention, immediate memory, long-term memory, working memory, and verbal fluency. No further significant improvement was observed between 12- and 65-months.^21^ Similar results were obtained by Ohta et al., (2022), they investigated the effect on cognition using the MMSE. A significant improvement in MMSE scores was found one year after implantation compared to preoperatively, but no significant difference was found comparing MMSE scores one year after implantation and MMSE scores two years after implantation.^20^ Mosnier et al., (2018) investigated the effect of cochlear implantation on cognition in individuals with MCI up to 7-years after implantation. They demonstrated a low rate of progression to dementia in these individuals, as only 6% of the individuals with MCI before implantation progressed towards dementia.^23^

### Limitations of studying cognitive function in the CI population

It is important to take the possible risk of practice effects into account in these studies. A possible solution to minimize practice effect is the use of different versions of the cognitive assessment tool, e.g., use RBANS-H A and B versions. However, previous studies mentioned that the use of two RBANS-H versions is not sufficient to completely eliminate practice effects.^13^, ^17^ A study design including a control group which is assessed at similar timepoints as the intervention group is required to measure practice effect, and correct for it. From a scientific point of view, the ideal study design to investigate the effect of CI on cognition is a randomized control design. This means that CI candidates are randomly assigned to either the control or intervention group. Such a research design is far from ethical in this situation, as subjects who meet the criteria to receive a CI would then be excluded from the treatment. A more plausible solution is the use of a matched control group, where intervention and control individuals are matched for important characteristics such as gender, mean age, duration of formal education, cognitive functioning, etc., However, it remains difficult to recruit individuals in the control group given the positive effects of the CI. Some reasons why an individual may not receive a CI and might be included in the control group are the following: the subject is still on a waiting list or did not meet the reimbursement criteria for cochlear implantation, the participant has a health condition which does not allow anesthesia or the ear anatomy did not allow cochlear implantation, or simply because the individual does not want a CI or prefers not to undergo surgery.^13^, ^17^ Since reimbursement criteria differ across countries a multicenter study could be a solution. However, even in a multicenter study, it is difficult to recruit a control group of sufficient size.^17^

Although most studies indicate a positive effect of CI on cognition, it is difficult to predict to what extent the improvement in cognitive functioning is related to the CI itself or to the auditory rehabilitation that individuals receive after cochlear implantation, as this can also have a positive effect on cognitive functioning. In addition, cognition is a complex aspect to study, as it is influenced by a broad range of different factors.^24^, ^25^ Decline in cognitive functioning is not only present in individuals with a pathologic brain disease, but also in healthy elderly. The normal ageing process is associated with changes in certain cognitive abilities, such as executive function, processing speed, visuospatial, etc. Interindividual differences are present in these age-related cognitive changes, but normal ageing does not affect daily life. When studying cognition, especially in the elderly, it is important to consider and correct for these changes.^26^ Another important factor that influences cognition is education, previous research indicated that the number of years of formal education is positively associated with cognitive functioning later in life.^27^ Depression is associated with cognitive functioning; more severe depressive symptoms are linked to decreased cognitive performance. Some cognitive domains are more severely affected by depression, such as executive function, processing speed, etc. In addition, depression and dementia are interconnected with each other. Depression is a risk factor for dementia, but it is also part of the prodrome and early stages of dementia.^24^, ^28^ Other modifiable risk factors for dementia, which may thus affect cognition in general, are hypertension, smoking, obesity, diabetes, excessive alcohol consumption, social contact, etc. When studying the effect of certain interventions on cognition, it is important to take these factors into account.^11^, ^24^, ^25^

### Future perspectives on unravelling how CI affects cognitive function

An objective biomarker for cognitive performance in CI users will help in understanding the impact of CI on cognitive functioning. Currently, several studies focus on the use of Electroencephalography (EEG), i.e., resting state EEG and Evoked Response Potentials (ERP), in the screening of cognitive functioning and dementia.^29^, ^30^, ^31^ EEG has many advantages, it is non-invasive, cost-effective, accessible, and has a high temporal resolution. Several studies point in the direction of Cortical Auditory Evoked Potentials (CAEPs) as a potential indicator for early-stage cognitive impairment.^31^, ^32^, ^33^, ^34^ A meta-analysis indicated prolonged P300 latencies in Alzheimer’s disease and MCI patients compared to controls. In addition, smaller P300 amplitudes were observed in Alzheimer’s disease patients compared to controls.^35^ Furthermore, other ERP components elicited with auditory oddball paradigms showed prolonged latencies and decreased amplitudes in MCI patients and Alzheimer’s disease patients.^33^, ^35^, ^36^ Although several studies point in the direction of CAEPs as a potential indicator for cognition, it is important to consider that hearing loss and CI use drastically affects CAEP morphology.^37^, ^38^ Unfortunately, research investigating the effect of both hearing loss and cognitive impairment on CAEP morphology is rather limited.^32^ An additional difficulty comes in place when investigating CAEPs in CI users, as the implant can generate electrical artifacts that contaminate the cortical responses. Although several artifact removal techniques are developed, it remains difficult to completely eliminate the artifact and it complicates the study design and data processing.^39^

An interesting approach to avoid bias linked to the hearing itself and the impact of CI artifacts on the cortical responses might be the use of other sensory-evoked potentials as indicators for cognition. A few studies already indicated that visual ERPs have potential as cognitive markers.^29^, ^30^, ^40^, ^41^ Stothart et al., (2015) used a visual oddball paradigm to elicit visual P1, N1, and Mismatch Negativity (MMN) in healthy older adults, MCI patients, and Alzheimer’s disease. They found a significant reduction in N1 amplitude in both MCI and Alzheimer’s disease compared to control. Moreover, visual MMN amplitude together with P1 amplitude in response to the deviant stimuli was a significant predictor of MMSE score.^30^ A more recent study evaluated theta responses during auditory and visual oddball paradigms, they observed a decrease in theta power as the cognitive decline increased.^29^ In line with the auditory evoked potentials, the P300 evoked with a visual oddball paradigm indicated differences in MCI patients compared to controls.^40^, ^41^ These results indicate the potential of visual ERP as an indicator for cognition, and may subsequently be an objective marker to evaluate cognitive performance in CI users.

## Conclusion

CIs are used to treat hearing loss in individuals with severe-to-profound hearing loss for whom hearing aids do not provide sufficient benefit. Previous research indicated that peripheral hearing impairment is associated with an increased risk of cognitive impairment in older adults. Although there is no standardized study protocol to investigate cognition after cochlear implantation, a significant improvement in cognition is observed in the majority of studies one year after cochlear implantation. Several considerations must be made when studying cognition in adults with a CI, i.e., suitability of the cognitive test for a population with a hearing impairment, practice effects linked to repeated testing, confounding factors such as depression, age, etc. The identification of an objective marker of cognitive functioning will help unravel how cochlear implantation affects cognition. Some studies already indicated that ERPs might serve as such a marker.

## Funding

This work was supported by a Fonds voor Wetenschappelijk Onderzoek (FWO) Fundamental Research Project (Grant Number G042819N3).

## Declaration of competing interest

Dr. Van Rompaey reported grants from Med-EL and Cochlear during the conduct of the study paid to the hospital. Dr. Lammers reported travel expenses reimbursed by MED-EL outside the submitted work. No other disclosures were reported.
